# Depression Prevalence in Neuropathic Pain and Its Impact on the Quality of Life

**DOI:** 10.1155/2020/7408508

**Published:** 2020-06-16

**Authors:** Farah Cherif, Hela G. Zouari, Wissal Cherif, Monia Hadded, Majda Cheour, Rahma Damak

**Affiliations:** ^1^Faculty of Medicine of Sfax, Avenue Majida Bouleila, Sfax 3029, Tunisia; ^2^Physiological Investigations, Habib Bourguiba University Hospital, Route de L'Ain, Sfax 3000, Tunisia; ^3^Ibn Oumrane Psychiatry Department, Razi University Hospital, Manouba, Tunisia; ^4^Faculty of Medicine, Tunis El Manar University, Tunis, Tunisia; ^5^Pain Treatment Center, La Rabta, Rue Jbel Lakhdar, La Rabta Jebbari, Tunis 1007, Tunisia

## Abstract

**Introduction:**

The management of neuropathic pain remains complex, generally because of the psychiatric comorbidity that is often underdiagnosed. The objectives of our work were to determine the link between depression and the characteristics of NP on the one hand and quality of life on the other hand, in a sample of subjects consulting for neuropathic pain (NP) regardless of etiology.

**Methods:**

We conducted a cross-sectional study involving 61 neuropathic pain consulting patients in whom we assessed five parameters, namely, neuropathic pain based on DN4, pain intensity using EVA, anxiety, and depression according to the HADS and quality of life.

**Results:**

The study population mean age was 52.71 ± 14.29 years while the sex ratio (m/f) was 0.52. The neuropathic pain's most common etiologies were postherpetic pain, carpal tunnel syndrome, and diabetic neuropathy. Depression and anxiety prevailed by 65.6% and 73.7%, respectively. The quality of life was impaired with average SF-12 physical and mental scores of 33.76 ± 8.03 and 37.78 ± 11.52, respectively. The overall mean BPI score was 5.53 ± 1.76. Patients with high DN4 scores were significantly more depressed (*p*=0.025). A significantly positive association was found between the depression score and the pain intensity (*p*=0.001, *r* = 0.41). Depressed subjects had a poor quality of life according to SF-12 and BPI.

**Conclusion:**

Given the depressive comorbidity impact on the neuropathic pain components as well as the quality of life, screening for this comorbidity should be part of the baseline ND assessment.

## 1. Introduction

Pain is a reason for frequent consultations in medicine [1]. It is an “unpleasant sensory and emotional experience, associated with actual or potential tissue injury or described in terms suggestive of such injury,” IASP (International Association for the Study of Pain), 1979. From a classical viewpoint, there are two main types of pain depending on whether there is a nerve injury or not: the pain known as “excess of nociception” and neuropathic pain (NP).

Since 1994, NP has been defined by the IASP as “pain initiated or caused by primary injury or dysfunction of the nervous system.” It has been proposed more recently to define NP as “pain associated with an injury or disease affecting the somatosensory system” [2]. This entity has an estimated prevalence of 7% in the general population and involves one-third of the patients suffering from chronic pain [3]. Despite the improved knowledge of the underlying NP neurophysiological factors and the pharmacological advances, this entity remains difficult to handle. Psychiatric comorbidity is, in fact, one of the main causes explaining the NP management complexity. According to Averill et al. [4], depression stands out as one of the most common psychological disturbances of pain. However, this disorder remains most often underdiagnosed, delaying these pains' therapeutic management.

In this context, several research works have studied the link between NP and depressive disorders. The prevalence of depression is estimated to be around 30% for NP patients [5–7]. However, most of the work achieved in these studies was performed on specific populations of patients suffering from NP according to the etiological origin.

In addition, pain may also interfere with the lives of NP patients by altering their quality of life (QoL), generating disabilities that may make them end up as vulnerable excluded individuals from society [8]. According to the World Health Organization (WHO), QoL corresponds to an individual's perception of his place in life, in the context of the cultural and value system in which he lives, in relation to his objectives, his expectations, his standards, and his worries. This concept refers to multiple dimensions, both objective and subjective: the person's somatic state of health, his functional abilities, his somatic sensations, his psychological health state, his social status, and his relational environment. Improving the QoL is therefore one of the main objectives of any therapeutic management. Thus, the depressive comorbidity screening and management in the NP patient could reduce the NP impact on QoL.

In Tunisia, pain is not sufficiently taken into account in our care environments yet. Indeed, the majority of our health professionals are neither well informed about nor sensitized to pain and its management. In fact, there is only one pain treatment center (PTC) throughout the country that was created in 1996 at the Rabta Hospital in the capital Tunis to manage chronic rebel pain.

Since the studies on the QoL and depressive disorders in NP patient are disparate and data are scarce in our context, we set the objectives of determining the prevalence of depression in a sample of subjects followed up for NP, first, and then investigate the link between this psychiatric comorbidity and NP characteristics as well as the QoL in this population.

## 2. Methods

A multicenter cross-sectional study was conducted at the Rabta PTC in Tunis and the functional exploration department at Hbib Bourguiba Hospital in Sfax, over a four-month period extending from March to June 2015.

### 2.1. Study Population

We included patients treated for NP, aged between 18 and 80 years. They all have a Neuropathic Pain (NP4) questionnaire score that is equal to or greater than four.

A group of patients was excluded; it involved pregnant women, patients with a personal psychiatric record, and those who experienced traumatic events during the last three months (death of an ascending or descendant parent, announcement of a serious illness or prognosis reserved for a parent or in the patient, family/marital conflict (divorce, separation, or violence)/professional (such as harassment or conflict), rape, road accident, and major financial difficulties) as well as those who were under tricyclic antidepressants treatment, the dose of which is ≥75 mg/day.

Nonconsenting patients and those suffering from cognitive impairment were not included, either.

### 2.2. Measurement Tools

We developed a semistructured questionnaire to collect the participants' sociodemographic data and records.

We used a four-question Neuropathic Pain (NP4) questionnaire for the diagnosis. This is a recent, simple, and quick tool to go through the diagnosis. It consists of seven items on the pain clinical description and another three-item part for the physical examination. A threshold value of an overall score greater than or equal to 4/10 establishes the NP diagnosis. In this study, we used the validated Arabic version [9].

In order to evaluate the pain severity, we used the Visual Analogic Scale (VAS) [10]. The VAS is a 100-millimeter ruler, fitted with a sliding cursor on the side shown to the patient. The cursor moves along a straight line with one end corresponding to the “absence of pain” and the other to “maximum imaginable pain.”

The patient has to position the cursor along this line to better indicate his pain intensity. The doctor can see the other side that consists of graduations, too. The positioning of the cursor by the patient allows the doctor to read the pain intensity.

Reading the VAS allows the classification of the intensity according to the following interpretation:

  0: absence of pain
  1–4: low-intensity pain
  5–7: moderate-intensity pain
  >7: severe-intensity pain

For the depression screening, we used the Arabic version of the Hospital Anxiety and Depression Scale (HADS) [11]. This is a 14-item scale rated from zero to three. Seven questions relate to anxiety and the other seven to the depressive dimension. This scale, thus, makes it possible to obtain two scores (maximum score of each = 21). A score ≥10 defines an indisputable symptomatology.

The patients' quality of life was assessed through two scales: the Short Form 12-item survey (SF-12) and the Brief Pain Inventory (BPI) in their Arabic versions [12–14].

SF-12 is a shortened version of the SF-36 questionnaire containing its main items only in order to have a faster evaluation by reducing the number of questions to the third. It calculates two scores: a physical quality of life score and a mental quality of life score.

While the physical domain explores the functional capacity related to pain, the psychological field investigates the NP influence on the patient's morale, mood, and well-being.

We considered a threshold based on the averages of the physical and mental scores of the Tunisian general population [13]. Thus, a lower score than 47.8 ± 9.8 defined an abnormality in the physical domain and a lower score than 43.4 ± 10.1 indicated an impairment in the mental domain.

The Brief Pain Inventory (BPI), a concise pain self-administered questionnaire originally designed to assess cancer pain [15], is currently used for other pain conditions [16]. We used seven items to measure the interference level of the pain with a certain function (general activity, mood, walking ability, normal work, relationship with other people, sleep, and the joy of life) using an evaluation scale that goes from 0 (no interference) to 10 (complete interference) for each item. The overall score would be from zero to ten (adding the scores of the seven items and then dividing by seven).

The study was approved of by the Sfax Faculty of Medicine Committee. The participating patients were informed of the study purpose and we obtained their oral consent. All the subjects diagnosed with psychiatric disorders were advised to consult a specialist.

### 2.3. Statistical Analysis

The collected data were entered and analyzed using the Statistical Package for Social Sciences (SPSS) computer software (23^rd^ version). The continuous variables distribution was compared to a normal distribution using the Shapiro–Wilk's test.

We calculated absolute and relative frequencies (percentages) for the qualitative variables. We also estimated the means, medians, and standard deviations and determined the extreme values for the quantitative variables.

The study of the relationship between the qualitative variables was achieved by Pearson's chi-square test and Fisher's exact test. The study of the relationship between the quantitative variables was done by the Pearson correlation test “*r*,” the linear adjustment curves, the Student *t*-test, and the coefficient of determination “*R*^2^.” The comparison of the several averages was performed by the “ANOVA” test. The significance threshold was fixed to *p* < 0.05.

## 3. Results

### 3.1. Study Population

A total of 61 patients were included in our study among whom forty-three were recruited from the PTC and 18 were recruited from the Functional Exploration Service. The population consisted of 40 women and 21 men, with a sex ratio of 0.52. The group average age was 52.71 ± 14.29 years. 72.1% of the study population were married, and 75.6% had some type of education. In fact, 42% of the cases attended secondary education; 19%, the primary schools; and only 14%, the higher institutions. Twenty-four patients (39.3%) were active at the time of the study. In addition, forty-one of the patients (67.2%) were urban dwellers and twenty subjects were of rural origin. The socioeconomic level was average in 72% of the population, low in 25%, and high in 5% of the cases.

In terms of clinical characteristics, 67.21% of the patients had a medical record: 34% had high blood pressure and 34% suffered from diabetes. The psychiatric family record of depression was revealed in seven patients, which represented 11.5% of the population.

The NP characteristics of the study population are summarized in Table 1.

### 3.2. Depression and Anxiety Prevalence

The study population mean depression score was 9.67 ± 5.2. Forty patients (65.57%) had depressive symptomatology with a mean score of 12.9 ± 3.79 according to the HADS. Depression was severe in 10 patients (25%), was of mild intensity in 16 subjects (40%), and was of light intensity in 14 patients (35%). In addition, the prevalence of anxiety was estimated at 73.77% of the cases, with an average score of 11.3 ± 4.48. We also found out that depression was positively correlated with anxiety (*p*=0, *r* = 0.5).

### 3.3. Study Population Quality of Life

**Figure 1:**
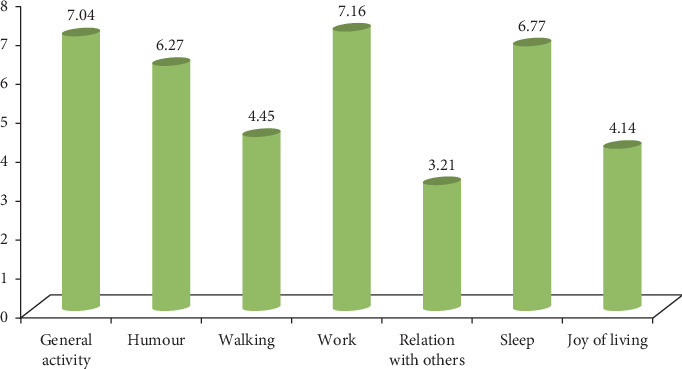
Repartition of the BPI items scores among the population.

According to the Short Form 12-item health survey (SF-12), the mean physical and mental scores were 33.76 ± 8.03 and 37.78 ± 11.52, respectively. The Brief Pain Inventory (BPI) scores had an average of 5.53 ± 1.76. The scores of the different dimensions are summarized in Figure 1.

### 3.4. Link between the Neuropathic Pain Clinical Features and Depressive Symptomatology

Relying on a dimensional approach, our study showed that depression is linked to etiology, intensity, and NP history (Table 2). In fact, it revealed a relationship between the severity of depression and that of NP by the VAS (*p*=0).

### 3.5. Link between Quality of Life and Depressive Symptomatology

According to SF-12, most of the depressed subjects were found to have a significantly affected quality of life both mentally (*r* = −0.46, *p*=0) and physically (*r* = 0.42, *p*=0.001).

Significant correlations were also found between the depression score and that of the total BPI and some interference items (Table 2). Patients with a severe depression had the most affected quality of life scores (Table 3).

### 3.6. Link between Sociodemographic Characteristics and Depressive Symptomatology

Our study also revealed a relationship between depression and a low socioeconomic level (*p*=0) and illiteracy (*p*=0.003). In addition, it reached no statistically significant correlations between depressive symptomatology and other sociodemographic features.

## 4. Discussion

In the present study, the prevalence of depression is found to be about 65.6%. Depression was positively correlated with the severity and duration of the NP treatment and a more affected QoL. Our results therefore highlight the link between NP and depression and the impact on the QoL.

It is worth noting that the revealed prevalence in our work was greater than that reported in the literature [5–7]. This prevalence was found to be 57.1% in a recent study involving patients with chronic pain followed up in PTCs in China [17]. The prevalence of depression in our study also exceeded that found in a similar study conducted in Tunisia in 2009. Cheour et al. found an estimated prevalence of 59% according to Beck's inventory [18]. However, this study included all chronic pain patients with no focus on the neuropathic part of the pain whereas we opted for involving NP patients only. This choice might partially explain our result. In fact, several studies [6, 19–21] have shown that the pain neuropathic component might be correlated with a greater prevalence of depression in pain patients. Furthermore, the high prevalence of depression in our study might be related to the nature of the study population. Most of our patients have actually been admitted to the PTC and are referred to because of their pain complexity and therapeutic resistance. This population is therefore at a greater risk of a depressive disorder [17]. This high prevalence could be evidence of a link between NP and depression. According to several recent research studies, these two features represent the somatic and psychiatric manifestations of the same neurobiological disorder [22, 23]. The pain endogenous regulation is achieved by endorphins that stimulate the neurons of the periaqueductal substance which, in turn, activates the neurons of the dorsal marrow containing serotonin. Once released, the latter inhibits transmissions to the medullary level [24]. The noradrenergic descending pathway inhibits the influx via the action of *α*^2^ adrenergic receptors, presynaptic affinity neuron, and postsynaptic neuron inhibitors of the dorsal horn of the spinal cord [25]. This pain-mediating system therefore uses the neurotransmitters (i.e., serotonin and norepinephrine), whose decrease is incriminated in depression. The dysregulation of these chemical mediators might then explain one of these pathologies or another and therefore a continuum between pain and depression. However, the various data described in the studies are still fragmentary and often phenomenological, but they illustrate the rapid progression of knowledge about the NP depressive consequences. Several other studies are still needed to refine these neurobiological hypotheses.

In our study, we also found a positive correlation between depression and pain intensity. This result is consistent with those reported in the literature where emotional distress has been found to vary with the pain severity [7, 26–28].

In practice, it is still difficult to establish the causality direction and chronology of the relationship between depression and NP intensity. On the one hand, studies have reported that depressive disorders can increase the pain sensation by lowering the threshold of pain perception [29] through neurobiological changes [29, 30]. Indeed, the brain areas related to a dysregulation of the mood are also the same structures involved in the modulation of pain. On the other hand, the intense NP would be at the origin of the depression symptoms and the intense moral suffering. Indeed, the NP has a nocturnal recrudescence that may explain its impact on sleep as forwarded by this study. This pain, especially the intense NPs, is also frequently associated with a restriction on interests and activities due to the physical disability it induces [31, 32].

Pain chronicity has also been associated with depression in the present study. As illustrated through the figures, patients with chronic NPs have a higher depression score than those with acute NPs (10 ± 5.33 versus 8.33 ± 4.97). However, this difference is not that significant. In a review by Bair et al., the presence of chronic pain was associated with the presence and severity of depressive symptomatology [23]. On the other hand, few studies have focused on acute NPs as they are generally regarded as a means of adaptation and protection [33].

However, we found a statistically significant positive correlation between treatment duration and the depression mean score (*p*=0.013, *r* = 0.31). The longer the duration of the treatment was, the more the patients were inclined to reveal depressive symptoms. Several explanations could be suggested to support this link. A long-term drug intake may have a heavy impact on the patient and become a source of psychological distress. In addition, the NP generally exhibits a partial response to analgesics, requiring the resort to therapeutic combinations, and therefore may expose the patient to a greater risk of adverse effects [34]. These conditions would consequently increase the emotional distress, especially depression. Incidentally, in our study, 65.85% of the treated patients received a therapeutic combination. Our result would also be related to past experiences our patients had gone through. Indeed, the majority of the included subjects were admitted to the PTC, after a long course of care during which they faced therapeutic failures. Such failures would worsen the emotional burden generated by the NP.

In our study the most depressed patients were those who had as their NP etiology a carcinological, poststroke, postoperative, or neuralgia V origin. To our knowledge, the link between depressive symptomatology and NP etiology has remained an unexplored territory. The study of the link between pain etiology and depression seems to be interesting since some pathologies could present an increased risk of depression. The aforementioned pathologies are thus responsible for emotional distress due to their disabling nature and the difficulty of their prognosis [35].

Another finding of our study is the significant link between depression and QoL of our pain-suffering patients according to the SF-12 scales. Our results, as well as those of the literature, have agreed on the deleterious effect of depression on the QoL dimensions and symptoms [36–38]. The study of the QoL dimensions by the BPI also confirmed this finding. Therefore, patients suffering from a severe pain and depressive symptomatology have an impaired walking capacity (*p*=0.002). In case the NP affects the lower limbs, it may result in a handicap for walking, which is added to the depressive symptomatology. Indeed, depression is characterized by motor functions slowdown and asthenia as well as somatic complaints. A clinical cross is therefore revealed between these two entities. This depression/NP crossover is also found for other BPI items in our study. Thus, NP patients with a severe intensity depression have high averages of pain interference in their “relationship with others” (*p*=0) and hence could be an important factor [39]. In addition, NP is often a pathology sequela (postherpetic pain, diabetes). The persistence of discomfort after “healing” may prolong the patient's mental suffering and may be difficult to understand. This incomprehension could also be manifested in the family. The absence of empathy toward this suffering and sometimes even the reproach caused by this complaint can feed the patient's depressive symptomatology and may even have repercussions on his relationships with others.

In addition, several authors have argued that the pain intensity would depend on the interaction between the cognitive, emotional, sociocultural, and physical factors [40] and would be strongly influenced, in addition to depression, by stress and anxiety.

This correlation, however, is to be taken with caution since the change in the relationship with the environment is at the heart of depression. The depressive symptomatology acts on the mood and is at the origin of an isolation of the suffering person.

In summary, our results emphasized the close relationship between NP, depression, and QoL of the pain sufferer. This relationship is to be taken into consideration when dealing with such patients. Indeed, several particularly NP promising psychosocial treatments including both cognitive restructuring [41, 42] and self-hypnosis training [43] can reduce this suffering and its negative impact on the QoL. It is worth noting that the QoL domains evaluated by the BPI are also related to each other. Consequently, any beneficial effect of the treatment on one domain will probably be accompanied by a more general improvement; for example, improving the NP patients' sleep or emotional functioning may also improve his physical functioning.

### 4.1. Study Limitations

Our research study may suffer some limitations. It might be criticised for involving NP patients who were admitted to the PTC and the Functional Investigation Service. Actually we chose these centers because of the varied population, compared to a population of a specific hospital service. Nevertheless, we have tried to limit the methodological bias throughout the study by recruiting patients whose NP is the main reason for consultation.

The second possible limitation is the reduced number of the involved patients in the study. This is a real weakness that limits the significance of some results and the scope of our interpretations. Therefore, our conclusions need to be confirmed by subsequent studies with a larger sample.

Third, it is important to point out that two-thirds of the patients were taking medication during the study. This means that drug effects on pain assessment and scaling results are possible. As a matter of fact, we considered it unethical to stop the drugs course for the exploratory purposes of our study.

Ultimately, the HADS we chose to evaluate the depressive symptomatology is a screening and not a diagnosis scale. An overestimation of this disorder is then possible in the absence of an associated psychiatric interview. However, this tool has been specially developed to avoid confusion related to the symptoms revealed in both depression and somatic disease cases. We also opted to this tool as we were motivated by its rapidity and ease of use.

## 5. Conclusion

Our results highlighted a high prevalence of depression among NP patients and its intricacy with the different NP components as well as its negative impact on QoL. In clinical practice, when we notice a significant alteration of an NP patient QoL, we should not be satisfied to attribute it to his pain or causal pathology. We should rather look for depression that could have aggravated this deterioration. Consequently, screening for depressive comorbidity should be an integral part of the baseline NP assessment. In this context, the HADS is an easy-to-use screening tool. Sensetizing the intervening parts on NP patients about the value of using this tool is also essential. In fact, the QoL assessment should also be part of the management of any pain sufferer as soon as the NP diagnosis is performed.

## Figures and Tables

**Table 1:** NP characteristics of the study population.

NP4 score	6.49 ± 1.54	

Pain etiology	Diabetic neuropathy	6
	Postherpetic pain	11
	Pain of rheumatic origin^*∗*^	32
	Others^*∗∗*^	12
	7.57 ± 1.73	

Pain intensity according to EVA	Moderate intensity	18 (29.5)
	Severe intensity	43 (70.5)
Pain history	2 years (min: 1 month; max: 25 years)	

Pain evolution	≥6 months	49 (80.3)
	<6 months	12 (12.7)

Treatment	Without treatment	20 (32.8)
	With treatment	40 (67.2)
Treatment duration	1.29 years (min: 0; max: 15 years)	

Number of treatments	Monotherapy	13 (32.5)
	Polytherapy (≥2 medicines)	27 (67.5)

Treatment nature	Morphine	3
	Tramadol	16
	Paracetamol	25
	NSAIDs	5
	Tricyclic antidepressants	17
	New antiepileptics	13
	Classical antiepileptics	3

Mean ± standard deviation; ^*∗*^lumbosciatic, cervicobrachial neuralgia, carpal tunnel syndrome, and polyradiculopathy; ^*∗∗*^stroke, postsurgery, oncological, neuralgia of 6. NP4: Neuropathic Pain 4 questionnaire; VAS: Visual Analogic Scale; min: minimal value; max: maximal value, number (%); NSAI: nonsteroidal anti-inflammatory.

**Table 2:** Link between depression and the characteristics of the neuropathic pain.

	Depression score	*r*	*p*
NP4 score	—	0.28	0.025^*∗*^
Diabetic neuropathy	12.67	—	0.001^*∗∗*^
Postherpetic pain	11.09		
Pain of rheumatic origin	7.31		
Others	13.17		
VAS score	—	0.41	0.001^*∗*^
Pain evolution		—	*NS* ^*∗∗∗*^
≥6 months	10 (5.33)		
<6 months	8.33 (4.9)		
Treatment duration	—	0.31	0.013^*∗*^
Mean BPI score	—	0.59	0
Activity	—	—	0.32
Humour	—	—	0.32
Walking	—	0.49	0
Steady work	—	—	0.16
Relation with others	—	0.5	0
Sleep	—	0.25	0.04
Joy of living	—	0.42	0.001

^*∗*^Student *t*-test; ^*∗∗*^ANOVA; ^*∗∗∗*^chi-square test; (): standard deviation. NP4: Neuropathic Pain 4 questionnaire; VAS: Visual Analogic Scale; NS: nonsignificant; BPI: Brief Pain Inventory.

**Table 3:** Link between depression intensity and QoL.

	Depression intensity				
	Absent	Light	Moderate	Intense	*p* ^*∗*^
SF-12 physical score	36.62 ± 8.4	31.19 ± 6	33.67 ± 7.2	27.28 ± 4.2	0.001
SF-12 mental score	43.25 ± 13	37.77 ± 12	36.37 ± 7	28.55 ± 6.3	0.007
BPI	4.5 ± 1.5	5.7 ± 1.3	5.36 ± 1.2	7.7 ± 1.4	0
Activity	6.66 ± 1.95	7.5 ± 1.78	6.62 ± 2.52	7.9 ± 2.1	NS
Humour	5.85 ± 2.7	6.92 ± 2.89	6 ± 2.3	6.7 ± 3.83	NS
Walking	2.95 ± 3.8	3.71 ± 3.1	4.68 ± 3.6	8.3 ± 2.5	0.002
Steady work	6.66 ± 2.3	6.85 ± 1.8	6.68 ± 2	8.5 ± 2	NS
Relation with others	1.95 ± 3.3	2 ± 2.8	3.8 ± 2.8	6.6 ± 3.8	0.002
Sleep	5.85 ± 3	7 ± 2.6	6.31 ± 3	9.1 ± 1.7	0.039
Joy of living	2.8 ± 3	4.9 ± 3.7	3.4 ± 2.2	7 ± 2.8	NS

^*∗*^ANOVA; mean ± standard deviation. SF-12: Short Form 12-item survey; BPI: Brief Pain Inventory; NS: nonsignificant.

## Data Availability

The data are available upon request to the corresponding author.

## References

[1] Van Hecke O., Torrance N., Smith B. H. (2013). Chronic pain epidemiology and its clinical relevance. *British Journal of Anaesthesia*.

[2] Merskey H., Bogduk N. (1994). *Classification of Chronic Pain: Descriptions of Chronic Pain Syndromes and Definitions of Pain Terms*.

[3] Bouhassira D., Lantéri-Minet M., Attal N., Laurent B., Touboul C. (2008). Prevalence of chronic pain with neuropathic characteristics in the general population. *Pain*.

[4] Averill P. M., Novy D. M., Nelson D. V., Berry L. A. (1996). Correlates of depression in chronic pain patients: a comprehensive examination. *Pain*.

[5] Gustorff B., Dorner T., Likar R. (2007). Prevalence of self-reported neuropathic pain and impact on quality of life: a prospective representative survey. *Acta Anaesthesiologica Scandinavica*.

[6] Radat F., Margot-Duclot A., Attal N. (2013). Psychiatric co-morbidities in patients with chronic peripheral neuropathic pain: a multicentre cohort study: psychiatric co-morbidities and neuropathic pain. *European Journal of Pain*.

[7] Mesci N., Mesci E., Külcü D. G. (2016). Association of neuropathic pain with ultrasonographic measurements of femoral cartilage thickness and clinical parameters in patients with knee osteoarthritis. *Journal of Physical Therapy Science*.

[8] Henschke N., Kamper S. J., Maher C. G. (2015). The epidemiology and economic consequences of pain. *Mayo Clinic Proceedings*.

[9] Terkawi A., Abolkhair A., Didier B. (2017). Development and validation of arabic version of the douleur neuropathique 4 questionnaire. *Saudi Journal of Anaesthesia*.

[10] Campbell W. I., Lewis S. (1990). Visual analogue measurement of pain. *The Ulster Medical Journal*.

[11] Terkawi A., Tsang S., AlKahtani G. (2017). Development and validation of arabic version of the hospital anxiety and depression scale. *Saudi Journal of Anaesthesia*.

[12] Younsi M. (2015). Health-related quality-of-life measures: evidence from Tunisian population using the SF-12 health survey. *Value in Health Regional Issues*.

[13] Younsi M., Chakroun M. (2014). Inequality and social heterogeneity in self-reported health status in the Tunisian population: an analysis using the mimic model. *Applied Research in Quality of Life*.

[14] Nejmi M., Wang X., Gning I., Mendoza T., Cleeland C. (2006). Validation de la version en arabe du BPI dans la douleur cancéreuse. *Courr Algol*.

[15] Cleeland C. S., Ryan K. M. (1994). Pain assessment: global use of the brief pain inventory. *Annals of the Academy of Medicine of Singapore*.

[16] Lantéri-Minet M. (2010). Les douleurs neuropathiques chroniques: diagnostic, évaluation et traitement en médecine ambulatoire: recommandations pour la pratique clinique de la société française d’étude et de traitement de la douleur. *Douleurs: Evaluation—Diagnostic—Traitement*.

[17] Wong W. S., Chen P. P., Yap J., Mak K. H., Tam B. K. H., Fielding R. (2011). Chronic pain and psychiatric morbidity: a comparison between patients attending specialist orthopedics clinic and multidisciplinary pain clinic. *Pain Medicine*.

[18] Cheour M., Ellouze F., Zine I., Haddad M. (2009). Évaluation de la dépression par l’inventaire de beck chez des patients souffrant de douleurs chroniques. *L’information Psychiatrique*.

[19] Aşkın A., Özkan A., Tosun A., Demirdal Ü., İsnaç F. (2017). Quality of life and functional capacity are adversely affected in osteoarthritis patients with neuropathic pain. *Kaohsiung Journal of Medical Sciences*.

[20] Uher T., Bob P. (2013). Neuropathic pain, depressive symptoms, and c-reactive protein in sciatica patients. *International Journal of Neuroscience*.

[21] Attal N., Lanteri-Minet M., Laurent B., Fermanian J., Bouhassira D. (2011). The specific disease burden of neuropathic pain: results of a french nationwide survey. *Pain*.

[22] Gambassi G. (2009). Pain and depression: the egg and the chicken story revisited. *Archives of Gerontology and Geriatrics*.

[23] Bair M. J., Robinson R. L., Katon W., Kroenke K. (2003). Depression and pain comorbidity. *Archives of Internal Medicine*.

[24] Basbaum A. I., Fields H. L. (1978). Endogenous pain control mechanisms: review and hypothesis. *Annals of Neurology*.

[25] Gaillard A. (2014). Douleur morale, douleur physique: mécanismes neurobiologiques et traitement. *Annales Médico-Psychologiques, Revue Psychiatrique*.

[26] Finan P. H., Buenaver L. F., Bounds S. C. (2013). Discordance between pain and radiographic severity in knee osteoarthritis: findings from quantitative sensory testing of central sensitization. *Arthritis & Rheumatism*.

[27] Hochman J. R., Gagliese L., Davis A. M., Hawker G. A. (2011). Neuropathic pain symptoms in a community knee oa cohort. *Osteoarthritis and Cartilage*.

[28] Gore M., Brandenburg N. A., Dukes E., Hoffman D. L., Tai K.-S., Stacey B. (2005). Pain severity in diabetic peripheral neuropathy is associated with patient functioning, symptom levels of anxiety and depression, and sleep. *Journal of Pain and Symptom Management*.

[29] Li J.-X. (2015). Pain and depression comorbidity: a preclinical perspective. *Behavioural Brain Research*.

[30] Stubbs B., Vancampfort D., Veronese N. (2017). Depression and pain: primary data and meta-analysis among 237,952 people across 47 low- and middle-income countries. *Psychological Medicine*.

[31] Poole H., Bramwell R., Murphy P. (2006). Factor structure of the beck depression inventory-II in patients with chronic pain. *The Clinical Journal of Pain*.

[32] Lempérière T., Programme de Recherche et d’Information Sur la Dépression (2003). *Dépression et Comorbidités Somatiques*.

[33] Morrison I., Perini I., Dunham J. (2013). Facets and mechanisms of adaptive pain behavior: predictive regulation and action. *Frontiers in Human Neuroscience*.

[34] Finnerup N. B., Attal N., Haroutounian S. (2015). Pharmacotherapy for neuropathic pain in adults: a systematic review and meta-analysis. *The Lancet Neurology*.

[35] Polsky D., Doshi J. A., Marcus S. (2005). Long-term risk for depressive symptoms after a medical diagnosis. *Archives of Internal Medicine*.

[36] Dermanovic V., Hrabac P., Skegro D. (2014). The impact of neuropathic pain and other comorbidities on the quality of life in patients with diabetes. *Health Qual Life Outcomes*.

[37] Schram M., Baan C., Pouwer F. (2009). Depression and quality of life in patients with diabetes: a systematic review from the European depression in diabetes (EDID) research consortium. *Current Diabetes Reviews*.

[38] McCollum M., Ellis S. L., Regensteiner J. G., Zhang W., Sullivan P. W. (2007). Minor depression and health status among US adults with diabetes mellitus. *American Journal of Managed Care*.

[39] Hendler N. (1984). Depression caused by chronic pain. *The Journal of Clinical Psychiatry*.

[40] Torta R. G. V., Munari J. (2010). Symptom cluster: depression and pain. *Surgical Oncology*.

[41] Knoerl R., Lavoie Smith E. M., Weisberg J. (2016). Chronic pain and cognitive behavioral therapy. *Western Journal of Nursing Research*.

[42] Jones R. C. W., Lawson E., Backonja M. (2016). Managing neuropathic pain. *Medical Clinics of North America*.

[43] Jensen M. P., Hanley M. A., Engel J. M. (2005). Hypnotic analgesia for chronic pain in persons with disabilities: a case series abstract. *International Journal of Clinical and Experimental Hypnosis*.

